# Microfibrous Scaffolds Guide Stem Cell Lumenogenesis and Brain Organoid Engineering

**DOI:** 10.1002/adma.202300305

**Published:** 2023-09-08

**Authors:** Kaja I. Ritzau-Reid, Sebastien J.P. Callens, Ruoxiao Xie, Martina Cihova, Daniel Reumann, Lino Prados-Martin, Richard Wang, Axel C. Moore, James P.K. Armstrong, Juergen A. Knoblich, Christopher L. Grigsby, Molly M. Stevens

**Affiliations:** Department of Materials, Department of Bioengineering, Institute of Biomedical Engineering, Imperial College London, London SW7 2AZ, UK; Institute of Molecular Biotechnology (IMBA) of the Austrian Academy of Sciences, Vienna BioCenter (VBC), Vienna 1030, Austria; Department of Materials, Department of Bioengineering, Institute of Biomedical Engineering, Imperial College London, London SW7 2AZ, UK; Institute of Molecular Biotechnology (IMBA) of the Austrian Academy of Sciences, Vienna BioCenter (VBC), Vienna 1030, Austria; Department of Medical Biochemistry and Biophysics, Karolinska Institutet, Stockholm 171 77, Sweden

**Keywords:** bioengineering, lumenogenesis, melt electrospinning writing, organoids, scaffolds, stem cells

## Abstract

3D organoids are widely used as tractable in vitro models capable of elucidating aspects of human development and disease. However, the manual and low-throughput culture methods, coupled with a low reproducibility and geometric heterogeneity, restrict the scope and application of organoid research. Combining expertise from stem cell biology and bioengineering offers a promising approach to address some of these limitations. Here, melt electrospinning writing is used to generate tuneable grid scaffolds that can guide the self-organization of pluripotent stem cells into patterned arrays of embryoid bodies. Grid geometry is shown to be a key determinant of stem cell self-organization, guiding the position and size of emerging lumens via curvature-controlled tissue growth. Two distinct methods for culturing scaffold-grown embryoid bodies into either interconnected or spatially discrete cerebral organoids are reported. These scaffolds provide a high-throughput method to generate, culture, and analyze large numbers of organoids, substantially reducing the time investment and manual labor involved in conventional methods of organoid culture. It is anticipated that this methodological development will open up new opportunities for guiding pluripotent stem cell culture, studying lumenogenesis, and generating large numbers of uniform organoids for high-throughput screening.

## 1 Introduction

During development, embryonic stem cells (ESCs) undergo a complex choreography of multilineage differentiation, polarization, symmetry breaking, and axis formation. This sophisticated process of self-organization ultimately generates a blueprint for cells to adopt distinct fates during embryogenesis. One critical process of self-organization is the formation of lumens: hollow cavities formed by the coordinated polarization of cells and the subsequent formation of apical and basolateral membrane domains.^[1]^ Lumenogenesis occurs during the initial stages of embryogenesis, with the radial organization of epiblasts in the blastocyst, and is also the first step of organogenesis for hollow anatomical structures, such as the intestines, kidneys, blood vessels, lungs, and neural tube, which later forms the central nervous system.^[2,3]^ This intrinsic capacity for self-organization has also been observed with in vitro cultures of pluripotent stem cells, including ESCs and induced pluripotent stem cells. For instance, 3D aggregates of pluripotent stem cells known as embryoid bodies (EBs) can spontaneously break their symmetry, form polarized lumens, and undergo coordinated programs of morphogenesis.^[4–6]^ This process has been leveraged for the development of organoids; 3D multicellular models which recapitulate certain structural and functional features of in vivo organs. In 2013, Lancaster et al. reported the development of cerebral organoids, which have become an established tool for investigating human brain developmental and neurological disease in vitro.[7] However, there are a number of enduring challenges that currently limit the effectiveness and scope of organoid models. In particular, there is high intra- and interbatch variability in their growth and structure, as well as a chaotic spatial distribution of resulting tissue regions.^[8]^ In addition, organoid models require time-intensive, manual manipulation for culture and analysis, which restricts their scalability for high-throughput screening.

These limitations can potentially be overcome through the use of bioengineering methods that are capable of guiding cell organization and steering tissue growth in a programmable fashion.^[9,10]^ Biophysical cues in the extracellular milieu, such as topography or applied mechanical loading, are known to elicit cellular responses that can contribute to the fate of individual cells as well as their collective growth dynamics.^[11,12,13]^ This principle contributes to the guidance of tissue organization during organogenesis and has been used to direct morphogenesis in vitro by micropatterning biomaterial substrates that mimic organ boundaries.^[14–17]^ In particular, microfibrous scaffolds, which have been known to guide tissue growth for decades,^[18]^ have been investigated as a means of directing the 3D assembly of stem cells and guiding their development into organoids. Free-floating poly(lactic-*co*-glycolic acid) microfibers have been seeded with pluripotent stem cells and used to engineer elongated cortical brain organoids, with the increased surface-area-to-volume ratio reported to improve the consistency and size of the neuroectoderm.^[19]^ In a more recent study, 3D-printed polycaprolactone (PCL) honeycomb meshes were used to engineer cerebral organoid sheets, with the flattened geometry shown to reduce core necrosis.^[20]^ These recent studies clearly demonstrate how microfibrous scaffolds can be used to direct the culture of brain organoids. However, for these two studies, each scaffold produced a single organoid with the geometry used only to define the bulk macroscopic structure (i.e., elongation, flattening).

**Figure 1:**
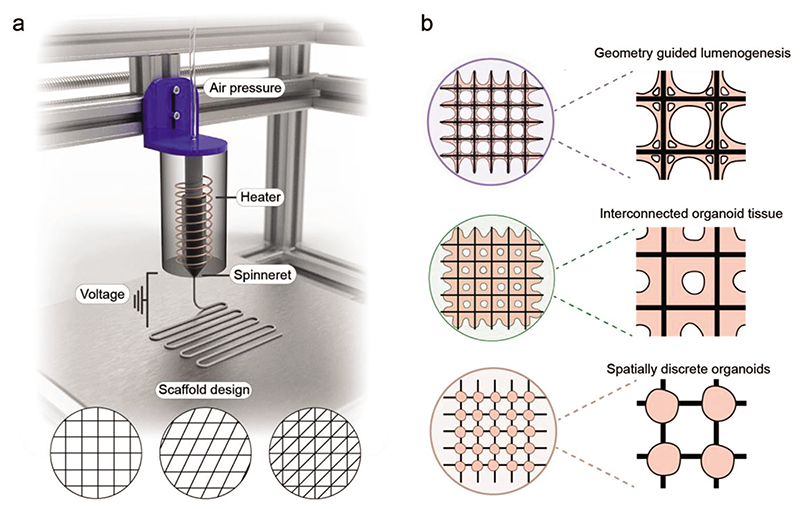
Scaffold-guided EB and organoid platform. a) Microfibrous scaffold fabrication using MEW. b) Schematic of MEW-fabricated scaffolds used to guide lumenogenesis and cerebral organoid growth. The cell–material interface of the scaffold is tuned to generate both interconnected and spatially discrete organoids.

We hypothesized that microfibrous scaffolds could be used to generate uniform and high-throughput arrays of brain organoids, with the geometry of the scaffold leveraged to direct cell assembly and lumenogenesis. In this study, we use melt electrospinning writing (MEW), a rapidly growing high-resolution fabrication technology with massive potential for porous tissue engineering scaffolds, to produce centimeter-sized microfibrous scaffolds (Figure 1a).^[21,22]^ Furthermore, we optimize a process workflow for uniform stem cell seeding and EB formation (Figure 1b). We show that the geometric parameters of the scaffold can be tuned to guide the emergence and morphology of lumens and differentiate these geometrically defined EBs into interconnected cerebral organoids. Finally, we demonstrate that by tuning the cell–material interface, we can generate high-throughput arrays of spatially separated cerebral organoids. Our microfabricated platform provides a new approach to cerebral organoid culture, offering key advantages in organoid uniformity, required skill level, and throughput, by eliminating some time-consuming and experimenter-dependent processing steps from traditional approaches. We envisage that these methods will provide new opportunities in engineering controlled and reproducible brain tissues for high-throughput modeling of neurological development and disease, while also offering a clear pathway to adapt this biotechnology for the culture of other organoid systems.

## 2 Results and Discussion

### 2.1 Fabrication of Uniform Grid Scaffolds Using MEW

We sought to establish a scaffold-based 3D culture platform capable of guiding stem cell assembly and organoid morphogenesis in a pre-determined and geometrically defined manner. Microfibrous grid-like scaffolds were fabricated using MEW, an extrusion-based 3D printing technology that enables layer-by-layer assembly of polymeric fibers. PCL was selected as a scaffold material: as a thermoplastic polymer with low melting temperature (≈60 °C) it is highly compatible with MEW,^[23,24]^ while its solid form is biocompatible with a slow biodegradation rate at ambient temperature.^[25]^ We fabricated 4 × 4 cm grid scaffolds with varying geometries, including square grids of different spacings, rhombus grids, and triangle grids (Figure S1a,b, Supporting Information). Each scaffold was fabricated in a layer-by-layer fashion, by stacking 10 layers of microfibers, resulting in a total height of ≈50 μm. To provide structural stability, the microfibers were interweaved at the intersections (i.e., in a “woodpile” fashion). SEM confirmed the deposition of homogenous, interwoven microfibers (diameter ≈ 5 μm) (Figure S1c, Supporting Information), while no scaffolds were ruptured in any of our experiments. The mean effective tensile modulus of the grid scaffolds (2–4 MPa) was consistent with the expected range for electrospun PCL,^[26]^ with values that could be tuned by adjusting the scaffold geometry (Figure S2, Supporting Information).

### 2.2 Grid Scaffolds Direct the Attachment and Growth Dynamics of Pluripotent Stem Cells

**Figure 2:**
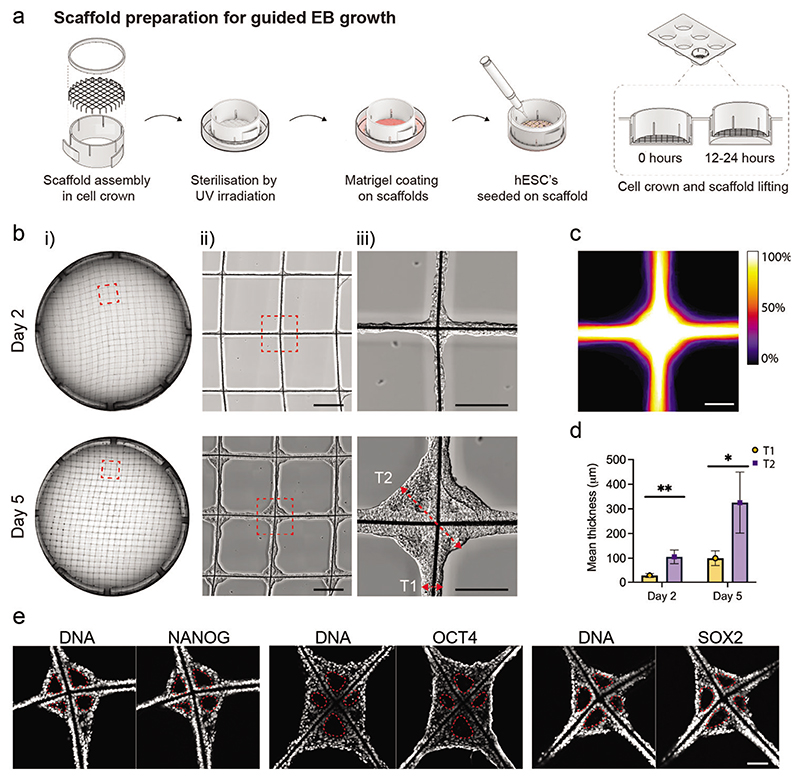
Scaffolds facilitate robust and reproducible control over EB self-organization. a) Schematic illustration of scaffold preparation for hESC seeding and EB tissue formation. b) Representative tiled overview images of the entire scaffold (i) and magnified views (ii,iii) on day 2 and day 5 after hESC seeding. A representative example of lumen emergence is shown on day 2 (black arrowhead) and an established lumen on day 5 (white arrowhead). The red dotted lines with arrow bars denote the region of tissue thickness measurement for the fiber walls (T1) and intersection (T2) on the scaffolds. Scale bars: (ii) 500 μm, and (iii) 200 μm. (*N* = minimum of 2 independent scaffolds). c) Heatmap showing superimposed bright-field images of individual intersections across the scaffold at day 5 after hESC seeding (*n* = 89 EB tissue nodes from 2 independent scaffolds). The color bar indicates the percentage of images in which tissue was present in these locations. Scale bar: 200 μm. d) Comparison of tissue thickness at the walls and intersections on day 2 and day 5. (*n* = 5 EB tissue nodes analyzed from 1 scaffold). Data represent mean ±standard deviation (s.d.) ** *p* < 0.01 and * *p* < 0.05, determined by Welch’s *t*-test. e) Characterization of EB tissue at day 5 by immunostaining with pluripotent markers SOX2, NANOG, and OCT4, and DAPI counterstaining of the nuclei (*n* = minimum of 3 EB tissue nodes on 1 or 2 independent scaffolds). The white dotted lines mark the lumen. Scale bar: 100 μm.

We optimized a protocol for seeding the MEW scaffolds with a uniform layer of hESCs (Figure 2a). Scaffolds were assembled within a cell crown to enable ease of handling during cell culture (Figure S3a, Supporting Information), and the microfibers were sterilized using UV irradiation. This design iteration involved treating the scaffolds with Matrigel basement membrane extract, which was visualized by SEM as a uniform coating dispersed across the microfibers (Figure S4, Supporting Information). hESCs (7 × 10^5^) were seeded onto the scaffold, which were allowed to adhere for 12 h before raising the scaffold to prevent further contact with the well surface. This resulted in widespread cell attachment, with bright-field imaging at day 2 and day 5 postseeding revealing a highly consistent distribution of hESCs and subsequent aggregated EB tissue across the scaffold (Figure 2b). The structural uniformity was evident from a heat map generated from a digital overlay of bright-field images displaying 89 different scaffold intersections on day 5 (Figure 2c). Interestingly, these bright-field images also revealed that the concave geometry provided by the scaffold intersections supported the growth of substantially thicker EB tissues than at the adjoining scaffold walls. Semi-quantitative analysis revealed that the tissue at the intersections was significantly thicker than at the scaffold walls (approximately a threefold increase) (Figure 2d). This observation is in line with previous studies of geometry-guided collagenous tissue growth, whereby the tissue has consistently been found to grow faster in concave scaffold regions, resulting in locally thicker tissues in those regions.^[27–30]^ Furthermore, the stemness of the tissues was validated by positive immunostaining for three pluripotency markers (SOX2, NANOG, OCT4) (Figure 2e). This expression profile was consistent with a pluripotent epiblast identity and suggested that the scaffold-cultured cells could be used to derive a plethora of different cell types to recapitulate features of human organogenesis.^[31,32]^

**Figure 3:**
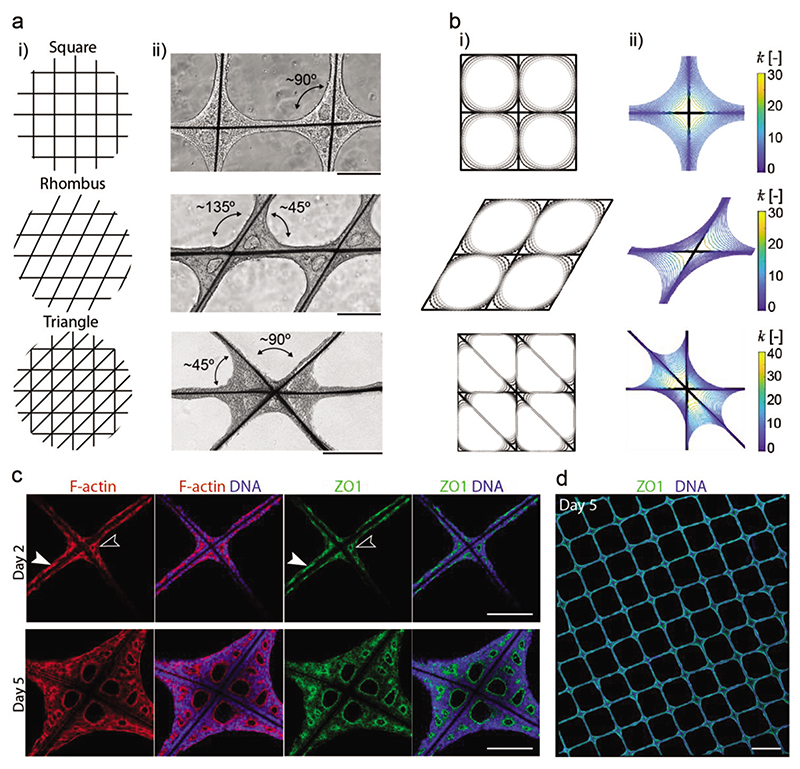
Scaffold geometry guides EB tissue formation. a) Graphical illustrations showing the different scaffold geometry designs investigated (i), bright-field images on day 5 showing EB tissue morphology on the different scaffold geometries (ii) on square grids that create four 90° angles as labeled in the bright-field image, rhombus grids that create two major angles of ≈135°and ≈45°, and triangle grids that create two major angles of ≈90°and ≈45°. b) Curve shortening flow models of EB tissue growth on scaffolds. Tissue growth evolution is modeled on: (i) square, rhombus, and triangle grid scaffolds, with lighter gray representing later time points, and (ii) evolution of normalized line curvature $\tilde{k}$ of the tissue interface. The key represents curvature. c) Characterization of lumen formation on square grid scaffolds at day 2 and day 5 by staining for F-actin (phalloidin), and immunostaining with apical protein marker ZO1 and nuclei (DAPI). A representative example of lumen emergence at the scaffold intersections is shown on day 2 (white line arrowheads) and at the scaffold walls (solid white arrowheads). The bottom row shows the matured tissue comprising the lumen at day 5 (*n* = minimum of 3 tissue nodes from 1 or 2 independent scaffolds). Scale bar: 200 μm. d) Tiled confocal fluorescence microscopy image of an immunostained scaffold sample with the apical protein marker ZO1, and counterstained with DAPI. Scale bar: 1 mm.

The observation that hESCs follow a curvature-driven tissue growth indicated that the EB tissue proliferation could be guided by tailoring the scaffold geometry. To probe this hypothesis, the growth dynamics of EB tissues were studied on grid scaffolds fabricated with square, rhombus, and triangular geometries (Figure 3a). These three designs retained the same number of nodes but provided a range of intersectional geometries, with square grids creating right angles (≈90°), rhombus grids generating acute and obtuse angles (≈45°, ≈135°), and triangular grids offering right angles adjacent to acute angles (≈45°, ≈90°). It was clear from bright-field microscopy that microfibers joined at ≈135° did not support outward tissue growth and resulted in largely underdeveloped tissues. Both the ≈45° and ≈90° angles supported the outward growth of EB tissue and the formation of lumens between the microfiber struts. Interestingly, the outward growth of the tissue at the ≈90° angle in the triangular grid was substantially reduced compared to the same angle in the square grid. This suggests competition in growth dynamics: while the square grid has four equivalent quadrants and largely symmetrical tissue proliferation, the triangular scaffold has acute angles favored for tissue growth at the expense of the outward growth of the adjacent ≈90° segments.

These results support the hypothesis that the scaffold geometry can be used to guide EB tissue growth. Curvature-controlled tissue growth has previously been described as a theoretical model to predict the collective growth behavior of cells in concave geometries.^[28]^ This model has been used to explain the growth dynamics of bone cells and fibroblasts that grow faster in concave regions^[33]^ which is consistent with our observations with hESCs. To study this growth behavior in silico, we implemented a simple 2D phenomenological growth model based on the mathematical concept of curve shortening flow theory (Figure 3b).^[34]^ In brief, this model describes the evolution of a closed contour (in this case representing the EB tissue surface), in which all points on the contour move in a normal direction at a speed proportional to their local curvature (Figure 3bii). Indeed, this simple model was able to accurately recapitulate the evolution of tissue growth that was observed empirically on the square and rhombus grids. This in silico model can be used to screen the expected behavior of tissue growth on a range of scaffold geometries, providing a programmable system that can be adapted to different applications for modeling organogenesis in vitro. Moreover, this phenomenological model could provide a starting point for more advanced (mechanistic) modeling strategies in future studies, taking into account cell growth and proliferation.

### 2.3 Scaffold-Guided Tissue Growth Directs Lumen Formation and Morphology

hESCs are known to intrinsically self-organize to form polarized epithelia with apical lumens,^[2,3,14,35]^ a trajectory that was observed in our scaffold cultures. Bright-field images captured on day 2 revealed epithelial polarization with the radial organization of hESCs. At day 5, the EB tissue had expanded, moreover, lumens were clearly present in each of the four corners of the scaffold intersections. To characterize lumen formation at days 2 and 5, we immunostained for the apical protein marker ZO1 and stained F-actin fibers using a phalloidin-based fluorescent dye (Figure 3c). On day 2, both ZO1 and F-actin were enriched at the scaffold corners, encircling a small emerging lumen, and also expressed along the scaffold walls. At day 5, ZO1 and F-actin staining revealed large primary lumens at the scaffold corners, flanked by secondary lumens that decreased in size with increasing distance from the intersection. These results clearly show that lumen expansion preferentially occurs at the concave regions of the scaffold. Tiled confocal imaging further demonstrated the reproducibility of this scaffold-guided lumen formation across the entire scaffold (Figure 3d). For example, a square grid scaffold (1000 μM wall-to-wall spacing) installed inside a 26-mm cell crown provides ≈460 scaffold intersections for tissue to grow and develop lumens. At 100% occupancy, this would result in ≈1840 polarized lumens growing in a highly organized way, highlighting the high-throughput potential of this platform.

**Figure 4:**
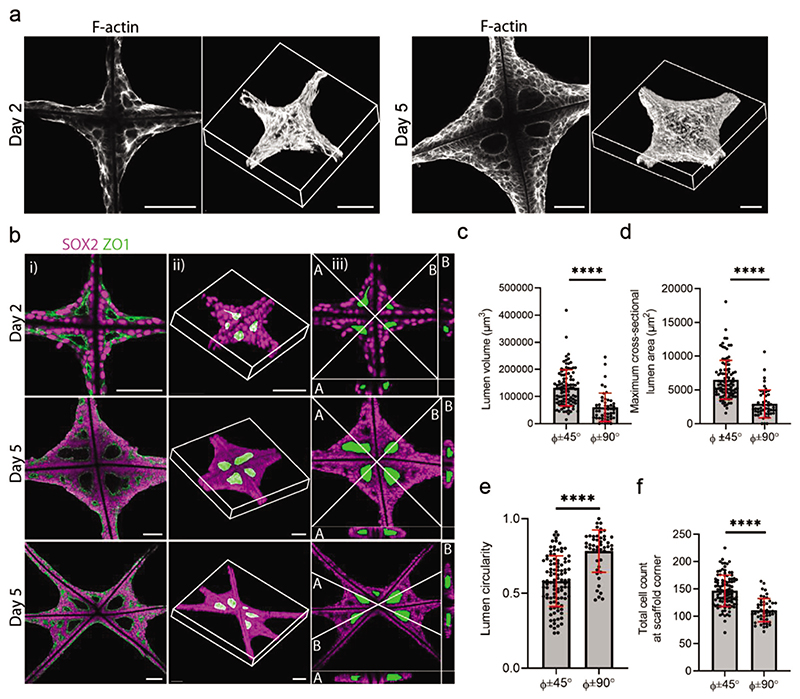
Scaffold geometry guides lumen formation. a) A single slice representation generated from confocal light microscopy (left) and 3D reconstructions (right) of F-actin stained samples on day 2 and day 5. Scale bar: 100 μm. b) Image gallery of a square grid scaffold on day 2 and day 5, and a triangle grid scaffold on day 5 showing a single slice of scaffold and EB tissue immunostained with SOX2 (magenta) and ZO1 (green) (i), 3D reconstruction from a confocal *z*-stack, with the lumens segmented and pseudo-colored in green (ii), cross-section of the confocal *z*-stack showing a bisector slice through the lumens indicated by the white lines (iii). Scale bar: 100 μm. c) Comparison between ≈45°angles (45°±9°) and ≈90°angles (90°±5°) on triangle grid scaffolds for lumen volume, d) lumen cross-sectional maximum area, e) lumen circularity, and f) total cell count per scaffold corner (*n* = 26 tissue nodes analyzed from *N* = 3 independent scaffolds). Φ represents the scaffold angle. Data represent mean ±s.d. **** *p* < 0.0001 as determined by Welch’s *t*-test.

We next sought to probe the volumetric structure of the EB tissue and lumens. For example, we reconstructed 3D confocal fluorescence microscopy images of EB tissues stained for F-actin on days 2 and 5 (Figure 4a). These images showed the EB tissue completely encompassing the scaffold, with a high density of cells at the central intersection that thinned toward the high-growth edges. This observation was validated by SEM performed on day 5 tissues (Figure S5a, Supporting Information). Higher magnification SEM images appeared to show elongated cells at the growth edge (Figure S5b, Supporting Information), indicating that the scaffold geometry promotes cell stretching between the angled scaffold walls. This finding further supported our hypothesis that the EB tissue behaves in a collective, curvaturedependent manner on the scaffolds. These SEM images also provided clear visualization of the lumen position and structure. Whilst the lumen is typically enclosed within the tissue by a polarized epithelium, the dehydration procedure caused shrinkage to the cells, thereby revealing a hollow lumen structure within the scaffold corner (Figure S5b, Supporting Information). The lumen structure was also readily identified when segmenting 3D confocal fluorescence microscopy stacks of square and triangle grid scaffolds immunostained for ZO1 and SOX2 (pluripotency marker) (Figure 4b). Digital cross-sections showed ZO1 delineating the boundary of large primary lumens located centrally in the tissue and enclosed by layers of SOX2^+^ pluripotent stem cells.

The image segmentation of the 3D confocal fluorescence stacks allowed semi-quantitative analysis of the large primary lumen (volume, maximum cross-sectional area, cross-sectional circularity) as well as the total cell count in each scaffold segment. All parameters were determined as a function of the angle of the scaffold walls surrounding the lumen in order to investigate whether the scaffold geometry might influence primary lumenogenesis. Significantly larger lumens (volume and maximum cross-sectional area) were observed in the EB tissue confined within ≈45° angles, compared to those within 90° angles (Figure 4c,d). In contrast, lumen circularity was significantly higher and more consistent in the EB tissues formed between ≈90° angles (0.83 ± 0.08), compared to those within ≈45° angles (0.70 ± 0.12) (Figure 4e). Finally, we observed a significant 26% increase in the number of cells present in the EB tissues within the ≈45° angles (152 ± 30) compared to those within the ≈90° angles (121 ± 21) (Figure 4f). Taken together, these results demonstrated a quantitative effect of the scaffold geometry on the morphological characteristics of the EB tissue, whereby the higher curvature offered by the ≈45° angles resulted in an increased cell density and lumens of greater size and reduced circularity.

### 2.4 Grid Scaffolds Support the Formation of Interconnected Cerebral Organoids

**Figure 5:**
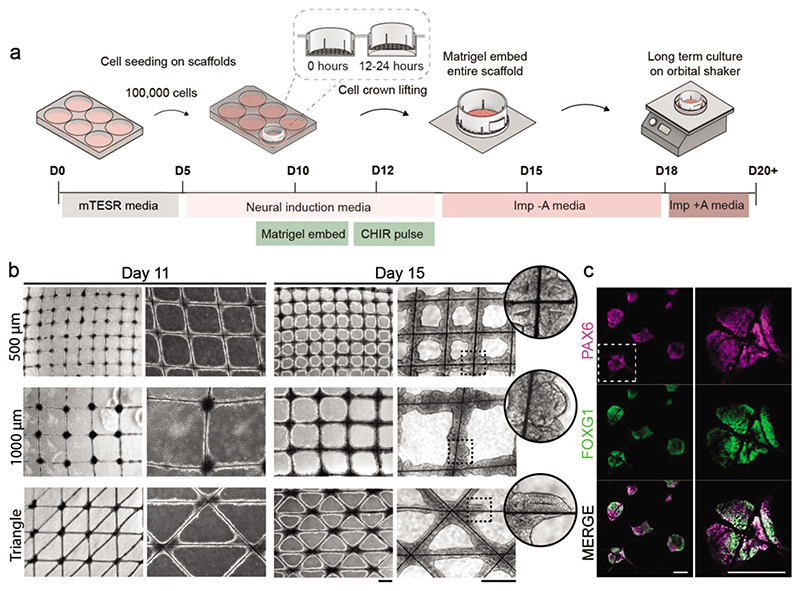
EB tissue forms cerebral organoids on scaffolds. a) Schematic illustration of adapted scaffold method for cerebral organoid generation. b) Representative bright-field images showing the development of cerebral organoids on scaffolds at days 11 and 15 on square grid scaffolds of 500 μM spacing, square grid scaffolds of 1000 μM spacing, and on triangle grid scaffolds. High reproducibility of the forming organoid tissue is apparent across all scaffolds. Scale bars: 500 μm. Magnified views of areas outlined by the black dotted lines are shown in circular insets. c) Representative images of histological sections of organoids on scaffolds at day 20 immunostained with the dorsal forebrain marker PAX6 (magenta) and the forebrain marker FOXG1 (green). The top row shows an overview of multiple organoids, and the bottom row shows a magnified view of the respective region marked with a white dashed line (representative images from 1 or 2 independent scaffolds). Scale bars: 200 μm.

Next, we investigated whether the EB tissue could be differentiated into cerebral organoids on the scaffolds. To this end, we cultured hESCs on square grid scaffolds (500 or 1000 μM spacing) or triangle grid scaffolds as described above, before implementing a widely used cerebral organoid protocol from day 5 onward (Figure 5a).^[36]^ Bright-field images showed that the tissue remained interconnected across all scaffolds and was largely homogenous in size, morphology, and density (Figure 5b). On day 11, tissue at the scaffold intersections appeared dense in the center with smooth, optically clear edges. This finding was consistent with the formation of neuroectoderm that is observed in conventional cultures of cerebral organoids.[7] These early-stage organoids were then embedded in Matrigel, which instigated a clear morphological change with the expansion of neuroepithelial buds evident by day 15. On days 15 and 17, the tissue on the scaffold walls appeared thicker on the 500 μM grid scaffolds compared to the 1000 μM grid scaffolds (Figure 5b; Figure S6, Supporting Information). This result can be phenomenologically explained by the fact that smaller scaffolds enable a larger proportion of cells to sense the curvature cues that promote tissue proliferation. This observation is consistent with findings from a recent study that reported that osteoblast cells reached confluence faster in scaffolds with smaller spacing (200 μm) compared to those with larger spacing (500, 600 μm).^[30]^ We next immunostained the scaffold-grown tissue to benchmark its cellular identity to literature reports of cerebral organoids grown using conventional methods (Figure 5c).^[7,37]^ Cells at the scaffold intersections expressed the anterior forebrain marker FOXG1, alongside the cortical progenitor marker PAX6. Co-expression of PAX6 and FOXG1 indicated widespread formation of cerebral tissue with a dorsal anterior forebrain identity.^[37]^ Furthermore, immunostaining for SOX2, a neural progenitor marker, revealed a thick layer of progenitor cells within a ventricular zone (VZ)-like region alongside cells expressing TUJ1, a marker for early neurons (Figure S7, Supporting Information). This finding indicated the occurrence of neurogenesis within these scaffold organoids.^[7]^ Together, these results show that our grid scaffold platform can be used to generate interconnected cerebral organoids with features that are consistent with conventional culture methods.

### 2.5 Grid Scaffolds Can Support High-Throughput Culture of Spatially Discrete Cerebral Organoids

**Figure 6:**
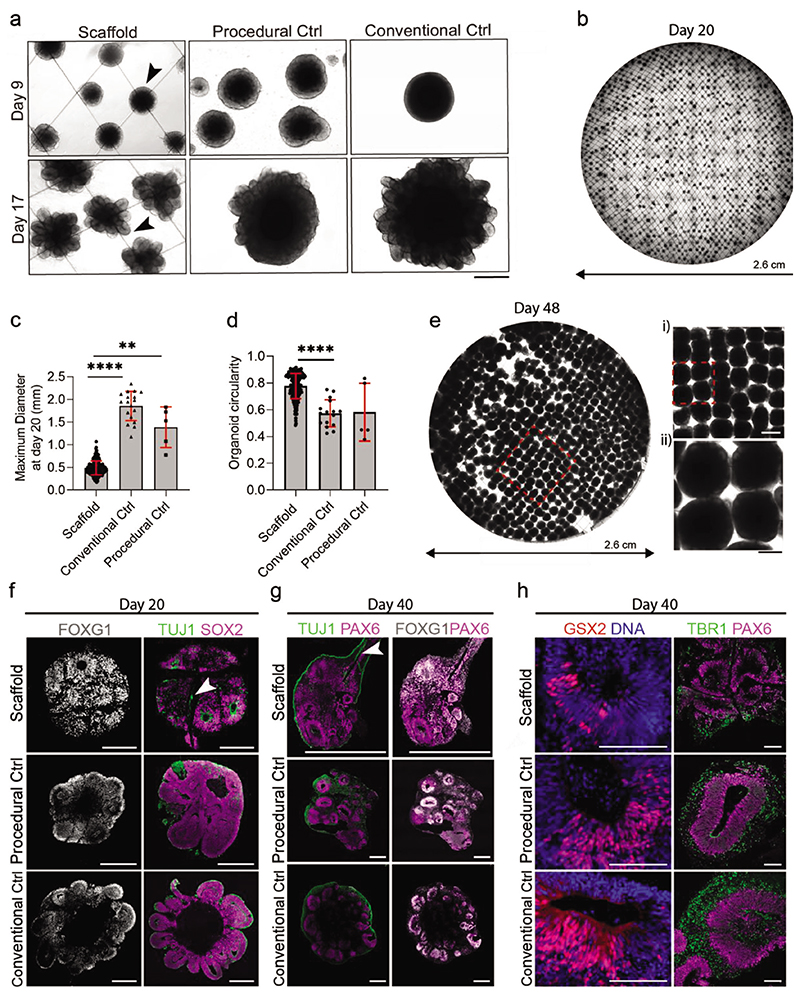
Spatially discrete cerebral organoids grow on uncoated scaffolds. a) Bright-field images show cerebral organoids grown on scaffolds, with procedural control organoids and conventional control organoids shown for reference. On day 9, before Matrigel’s addition, the smooth and optically translucent organoid edges are denoted by the black arrowhead. At day 17, after Matrigel addition and CHIR pulse, neuroepithelial buds are denoted by the black arrowhead. Scale bar: 500 μm. b) Representative tiled overview image of the entire scaffold with organoids at day 20 (*N* = 3 independent scaffolds). c) Maximum diameter of organoids at day 20. Analysis was performed on organoids using a custom Fiji macro (*n* = minimum of 5 organoids from 3 independent scaffolds). Data represent mean ±s.d. **** *p* < 0.0001 and ** *p* < 0.001, determined by Kruskal–Wallis with Dunn’s post-test. d) Comparison of organoid circularity at day 20 obtained through image analysis using the inbuilt measure function in Fiji (*n* = minimum of 5 organoids from 3 independent scaffolds). Data represent mean ±s.d. **** *p* < 0.0001, determined by Kruskal–Wallis with Dunn’s post-test. e) Representative tiled overview image of fixed organoids on 1000 μM spaced scaffolds at day 48. Magnified regions are marked by a red dashed line. Scale bars: 1000 μM (top) and 500 μM (bottom). f) Immunostaining characterization of day 20 cerebral organoids on scaffolds, compared to procedural and conventional control organoids. On the left, histological sections show organoids immunostained with forebrain marker FOXG1 (grey). On the right, histological sections show organoids immunostained with neuronal marker TUJ1 (green), and neural progenitor marker SOX2 (magenta) (*n* = minimum of 4 organoids). Scale bars: 200 μm. g) Histological sections of day 40 organoids immunostained with dorsal forebrain markers PAX6 (magenta), neuronal marker TUJ1 (green), and FOXG1 (white), Neurons are seen to grow inside the organoid along the scaffold fibers (white arrow). Scale bar: 500 μm. h) Histological sections of day 40 organoids immunostained with ventral marker GSX2 (red) and counterstained with DAPI (blue), and dorsal markers PAX6 (magenta) and TBR1 (green). Scale bar: 100 μm.

We next sought to use the grid scaffolds to generate discrete cerebral organoids that would more closely mimic the morphology of conventional organoid cultures. While Matrigel-coated scaffolds enabled widespread attachment and proliferation of stem cells, we reasoned that uncoated scaffolds would result in spontaneous cell aggregation at the scaffold intersections. Indeed, bright-field microscopy showed the pluripotent stem cells clustering at the scaffold intersections by day 2 (Figure S8, Supporting Information). The scaffold-bound organoids exhibited smooth edges and optically translucent borders on day 9, with neuroepithelial budding clearly present on day 17 (Figure 6a). These morphological features were consistent with organoids grown using conventional protocols (EBs aggregated in a 96-well plate) and a procedural control (single cell suspensions in a 6-well plate, without a scaffold present). Similarly to the Matrigel-coated scaffolds, these spatially discrete organoids were Matrigel-embedded when signs of optical clearing at the organoid borders were clearly present. Our scaffold platform provides a method to simultaneously embed large quantities of organoids in a simple, single-step process. This method offers considerable practical advantages when compared to the embedding of free-floating organoids, which is manual, time-intensive, and low-throughput.

Bright-field images at day 20 revealed a highly ordered array of uniform organoids distributed across the entire scaffold (Figure 6b). To assess the efficiency of organoid formation on the scaffolds, we measured the percentage of intersections that were occupied by an organoid. In a typical square grid with 500 μM spacing, the occupancy was 35 ± 14% (402 ± 161 organoids per scaffold). These results demonstrate that while the organoid generation on the scaffolds is high-throughput, there remains significant potential to improve occupancy. The scaffold-cultured organoids had a maximum diameter of 0.47 ± 0.15 mm at day 20, which was significantly smaller than both the conventional cultures (1.95 ± 0.33 mm) and the procedural controls (1.53 ± 0.45 mm) (Figure 6c). The scaffold-cultured organoids also showed a significantly higher circularity (0.78 ± 0.09) than the conventional cultures (0.57 ± 0.1) (Figure 6d). After ≈40 days of culture, however, some scaffolds exhibited areas of uncontrolled growth between adjacent organoids, which could potentially be due to local Matrigel coating of the scaffold microfibers at the embedding stage. In addition, by day 40, the organoids had outgrown the 500 μM spaced grids, leading to organoid fusion. To enable the long-term culture of spatially discrete organoids, we further adapted the protocol by using 1000 μM spaced grid scaffolds and replacing the embedding step with 2% Matrigel supplemented in the culture media. Tiled bright-field images at day 48 of this revised protocol qualitatively revealed that the scaffolds were well populated with discrete organoids (Figure 6e), while immunostaining confirmed the presence of VZ-like regions with expression of FOXG1 and MAP2 (neuronal marker) (Figure S9, Supporting Information). This revised protocol, which was streamlined due to the removal of the Matrigel embedding stage, was used for all subsequent studies.

We compared neurodevelopmental timings and identities of scaffold-derived organoids with conventional and procedural controls and did not find substantial differences in tissue identity as well as neural differentiation in all three groups. At day 20, we observed expression of the forebrain marker FOXG1, sparsely distributed TUJ1^+^ neurons, as well as the presence of the SOX2^+^ neural progenitors in the VZ-like lumen regions (Figure 6f). Regional co-expression of FOXG1 and PAX6 indicated a dorsal forebrain identity at day 40 (Figure 6g) and day 48 (Figure S10a, Supporting Information). Further evidence for this regional identity was offered by the widespread expression of the deep layer subcortical dorsal marker TBR1 and low expression levels of the ventral forebrain marker GSX2 at day 40 (Figure 6h). Furthermore, we observed co-expression of the deep cortical marker CTIP2 and the neuronal marker MAP2 at day 48 (Figure S10b, Supporting Information). For longer-term organoid culture, the scaffold grid spacing was increased to 4000 μM to accommodate organoid growth and avoid organoid fusion. After 60 days of culture, organoids showed large VZ-like regions with dorsal forebrain identity, abundant CTIP2+neurons in the deep cortical layers, and MAP2+neurons on the outer cortical layers (Figure S8c,d, Supporting Information). Overall, the expression, timing, and distribution of key markers were largely consistent with the conventional cultures and procedural controls, indicating that the grid scaffolds were able to guide cell assembly without interfering with subsequent biological development. However, there were a few notable observations. At days 20 and 40, TUJ1+neurons were observed at the interface between the scaffold and the center of the organoid. Previous research has shown that micropatterned scaffolds can provide axonal guidance cues and guide regional cellular identity and migration.^[38,39,40]^ These results suggest that scaffold fibers could provide structural guidance for neurons, offering the possibility that scaffolds may be harnessed to specifically direct neural migration and axon outgrowth in future work.

## 3 Conclusion

We have developed a biomaterial platform to guide stem cell selforganization and generate high-throughput, scaffold-cultured organoids. We used scaffold geometry to modulate lumen formation in a reproducible and programmable way, which will provide opportunities to study lumenogenesis in a high-throughput manner. Moreover, we believe that this work could also be applied to guided brain organoid protocols, to generate regionspecific organoids. For example, to study the effects of geometric guidance and shape restrictions on the development of neuroepithelium, which is relevant in the context of neural tube patterning (and thus the generation of different brain regions) as well as neural tube defects. We next showed that the scaffold platform can support the growth of interconnected cerebral organoid tissues in a highly controlled manner. This approach offered several key practical advantages, such as immobilizing the organoids in a fixed position for Matrigel embedding and bioimaging, a feature that would also be highly beneficial for other studies, such as optogenetics, light-inducible CRISPR, and experiments requiring modification of individual organoids. We next tuned the cell–material interface to form arrays of spatially discrete EBs that we used to derive hundreds of independently growing cerebral organoids that could be cultured, treated, and imaged en masse. Crucially, the scaffold grid spacings could be increased to accommodate the long-term culture of cerebral organoids that exhibited more mature phenotypic features. These initial results are promising and warrant further optimization and characterization of the platform, for example, to obtain deeper insight into the potential formation of necrotic cores or the ability to sustain even longer organoid culture.

Taken together, this approach addresses some outstanding technical challenges in the field, namely the labor-intensive culture methods involving highly repetitive manual actions, and the resulting low-throughput culture. Our platform offers increasing handling ability that can drastically expedite culture protocols (e.g., feeding and Matrigel embedding) and subsequent characterization (e.g., time-lapse and high-throughput imaging). The application of bioengineering for organoid culture requires a fine balance between guiding the tissue growth using external cues and simultaneously facilitating the stochastic nature of selforganization that is key to organogenesis. We believe that this platform achieves this goal, providing the field with an accessible culture system with enormous potential for high-throughput screening and adaptability for other organoid systems, such as cardioids or intestinal organoids.

## 4 Experimental Section

### Melt Electrospinning Writing for Scaffold Fabrication

Scaffolds were fabricated using a custom-built MEW machine purchased from the Hutmacher group at Queensland University of Technology (Brisbane, Australia).^[23]^ The machine contained a controllable stage, a heating chamber with two separately controllable heating elements, an electropneumatic pump to control the extrusion pressure, and an electrode that was connected to a high-voltage source. A 50–50 PCL blend of *M*_w_ ≈ 80 000 g mol^−1^ PCL (Sigma, 440744) and *M*_w_ ≈ 45 000 g mol^−1^ PCL (Sigma, 704105) was loaded into a 3 cc polypropylene syringe (Nordson #7012074) and heated to 70 °C for 30 min. A 23 G needle (Nordson #7018302) was installed, and the syringe was inserted into the spinneret. PCL scaffolds were printed using the following parameters: a 10 kV accelerating voltage, 10 mm collector distance, axis velocity of 1500 mm min^−1^, feeding air pressure of 1.0 bar, and heating temperatures between 60 and 80 °C. Electrospinning was conducted at room temperature condition (nominal temperature at 24 °C and humidity at 35%). Scaffold geometry was designed using CAD software (Rhino 3D), which was exported to a custom Python script that generated the corresponding G-code. The printing was controlled by MACH 3 CNC software (ARTSOFT, Livermore Falls, USA). Each scaffold consisted of 10 stacked layers. Scaffolds were detached from the collector plate using a drop of 100% ethanol and moved to a Petri dish. For all cell culture experiments, scaffolds were sterilized under UV light irradiation for 30–45 min (254 nm, 30 W source).

### Tensile Testing

Scaffold samples were cut to a size of 20 × 35 mm. Tensile testing was performed using a Bose Electroforce 3200 (BOSE, USA) with a 250 g load cell. Samples were clamped between parallel steel plates (grips). Double-sided tape was used on one side of the grip to assist in alignment and also provided an elastic foundation that prevented slipping. The stage was ramped from 0 mm to maximum stage travel 6.2 mm or failure, at a displacement rate of 0.1 mm s^−1^. The effective tensile modulus was calculated using a custom Matlab code. The code found the cycling region (10 cycles), extracted cycles 5–10, and fitted a linear regression. Experiments were carried out five times (*N* = 5).

### Scanning Electron Microscopy

MEW PCL scaffolds were cut with micro scissors and mounted on adhesive carbon tape on a scanning electron microscopy (SEM) stub. Prior to imaging, samples were gold sputter coated using an Emitech K575X sputter coater for 30 s at a deposition current of 20 mA. Images were obtained using a JSM 6010LA SEM (JEOL) at 20 kV acceleration voltage.

### Ethics

H9 human embryonic stem cells (hESCs) (WA09) were obtained from the WiCell Research Institute (USA), in compliance with the relevant ethical regulations and with donor-informed consent (NIHhESC-10-0062). The work was approved by the Steering Committee for the UK Bank and for the Use of Stem Cell Lines and adhered to the regulations of the UK Code of Practice for the Use of Human Stem Cell Lines.

### Cell Culture

H9 hESCs were obtained from WiCell and maintained in feeder-free culture conditions with verified normal karyotype. Cells were cultured on hESC-qualified Matrigel (diluted in DMEM/F12 according to manufacturer’s instructions) (Corning, cat. No 354 277) coated 6-well culture plates with 2 mL mTESR1 (Stemcell Technologies) media, which was exchanged daily. Cells were passaged every four to five days at 60–70% confluency by EDTA treatment. Cells were maintained in a 5% CO_2_ incubator at 37 °C. All cell lines were routinely tested for mycoplasma (on a monthly basis) by collecting spent culture medium and sending samples to an external company (Eurofins Genomics), and all tested negative.

### Scaffold Preparation for Cell Culture

PCL scaffolds were secured onto sterile CellCrown holders (Scaffdex CellCrown, Tampere, Finland), followed by sterilization with UV light irradiation for 30–45 min. Scaffolds were then stored in a sterile 6-well plate until cell seeding.

### Lumen Morphogenesis Protocol–Scaffold Preparation

Sterile scaffold-CellCrown assemblies were placed in 6-well plates and coated in 2 mL hESC-qualified Matrigel (diluted in DMEM/F12 according to manufacturer’s instructions) (Corning 354 277) and left to incubate at 37 °C for 1–2 h. Immediately before cell seeding, scaffolds were washed once in phosphate buffered saline (PBS).

### Day 0—Seeding onto Scaffolds

At ≈60% confluency, hESCs were dissociated from the well-plate by adding 1 mL accutase for 3 min at 37 °C. The remaining adherent cells were sprayed off with 1 mL mTESR (Stemcell Technologies) and cells were centrifuged at 200 g for 3 min. The cell pellet was resuspended in mTESR media and a cell suspension was prepared in mTESR media containing 50 μM ROCK inhibitor Y27632 (Selleckchem) and 1% (v/v) antibiotic–antimyocytic (ThermoFisher) at a density of 7 × 10^5^ cells per scaffold. After Matrigel incubation, scaffolds were washed once in PBS and placed in low attachment 6-well plates, or plates treated with anti-adherence rinsing solution (Stemcell Technologies) for 1 min, followed by washing three times in PBS. Scaffolds were secured at the bottom of the plate by pushing the CellCrown down using forceps. Cell suspension (3 mL) was added per well, and the plate was gently rocked in orthogonal directions to evenly distribute the cells across the scaffold.

### Day 1

After 12–24 h of culture, CellCrowns were lifted by ≈5 mm in the well plate to prevent cells from contacting the bottom of the well, while ensuring sufficient media coverage across the scaffold. The cells were left unperturbed on day 2, or fixed for 1 h at room temperature or overnight at 4 °C with 4% paraformaldehyde (PFA) (v/v) in PBS and washed three times in PBS. The PFA solution was commercially obtained (catalog 15700, Electron Microscopy Sciences).

### Day 3

mTESR media was exchanged without ROCK inhibitor or Antibiotic–Antimyocytic.

### Day 5

Cells were fixed as previously described. For the generation of cerebral organoids on scaffolds, media was exchanged with a neural induction media.

### Cerebral Organoid Media Formulations—Neural Induction Media

DMEM/F12 media (Gibco) was combined with 1% (v/v) N2 supplement, 1% (v/v) GlutaMAX Supplement (Invitrogen), 1% (v/v) MEM-NEAA (Invitrogen), 1% (v/v) penicillin/streptomycin, and 1 μg mL^−1^ heparin (Sigma, H3149). Sterile filtered before use.

### Improved — A Media

A mixture of DMEM/F12 (Gibco) and Neurobasal medium (Gibco) (1:1) containing 1% (v/v) B27 minus vitamin A, 1% (v/v) GlutaMAX supplement (Invitrogen), 0.5% (v/v) N2 supplement (Invitrogen), 0.025% (v/v) insulin (Sigma, I9278), 1% (v/v) penicillin/streptomycin and 1 mg mL^−1^ sodium bicarbonate (cell culture grade). Sterile filtered before use.

### *Improved* + *A Media*

A mixture of DMEM/F12 (Gibco) and Neurobasal medium (Gibco) (1:1) containing 1% (v/v) B27 plus vitamin A, 1% (v/v) GlutaMAX supplement (Invitrogen), 0.5% (v/v) N2 supplement (Invitrogen), 0.025% (v/v) insulin (Sigma, I9278), 1% (v/v) Vitamin C solution (from a stock solution of 7 mg mL^−1^ in DMEM/F12), 1% (v/v) penicillin/streptomycin and 1 mg mL^−1^ sodium bicarbonate (cell culture grade). Sterile filtered before use.

### Cerebral Organoid Culture for Conventional Controls

Cerebral organoids were generated as previously described with CHIR supplementation.^[19,24,25^] Briefly, 2 days after Matrigel embedding (typically day 13), 3 μM CHIR 99 021 (Tocris) was added to the neural induction media for 3 days in improved differentiation media −A. Four days later, improved +A media was added and media was changed every 2 or 3 days. At day 20, organoids were placed on an orbital shaker, at a speed of 57 rpm.

### Cerebral Organoid Formation on Scaffolds

PCL scaffolds were prepared and seeded with hESCs as previously described. For long-term organoid culture, 1000 μM scaffolds were used. On day 5, mTESR1 media was replaced with neural induction media. Media was replaced every day until days 9–12, when optical clearing on the organoid borders was clearly present. At this stage, CellCrowns (with scaffolds and organoids attached) were gently lifted from the 6-well plates and placed on parafilm in a 10 cm Petri dish. Matrigel was added to the scaffold, to completely cover the organoids (≈1 mL) and polymerized at 37 °C for 30 min in an incubator. For the revised protocol, this step was substituted by the addition of 2% Matrigel into the media, which was recently identified as a suitable alternative to solid embedding in Matrigel.^[26]^ The CellCrown was then gently removed from the parafilm (it was found that sliding the scaffold off the parafilm rather than lifting minimized any damage to the scaffold or organoids). The CellCrown and scaffold assembly was then placed in a new 6-well plate and filled with 4 mL of neural induction media. Two to three days after the first addition of Matrigel, the media was changed to Improved –A media. For 3 days, 3 μM CHIR 99021 (Tocris) was also added. Four days later, improved +A media was added and media was changed every 2 days. At day 15, the scaffolds were transferred to a 6 cm Petri dish to provide a higher volume of media (10 mL) to support organoid growth. The CellCrown was placed on a sterile plastic ring to prevent the organoids from touching the bottom of the plate. For spatially separated cerebral organoid culture, scaffolds were prepared as described, without the Matrigel coating step.

### Cerebral Organoid Culture for Procedural Controls

The same organoid culture procedure was followed as with scaffold-cultured cerebral organoids, with the omission of the cell crown and scaffold constructs. Organoids were Matrigel embedded using the same protocol as described for a conventional cerebral organoid generation.^[19,24,25]^

### Matrigel Supplementation Protocol

When signs of optical clearing on the organoid borders were clearly present, 2% (v/v) Matrigel (Corning, cat. no. 356235) was added to the neural induction media, and at every media change until the end of the culture period. The Matrigel solution was prepared by adding Matrigel to cold media. Matrigel was freshly added to cold media and warmed for 10–15 min at room temperature before addition to the organoids. This procedure avoided premature Matrigel polymerization and thermal shock to the cells.

### Bright-Field Imaging

Bright-field images were acquired with an EVOS XL Core imaging system (Thermo Fisher Scientific). For tiled images of the entire scaffold, images were acquired using a CellDiscoverer 7 (Zeiss) microscope using an AxioCam702 (mono) and a Plan-Apochromat 5 × 0.35 NA objective, and with Zen Blue 2 software (Zeiss). To adjust for drift, *z*-stacks (25 × 27 μm) were recorded. Tiled images were fused using the Zeiss image processing software. To quantify the thickness of the tissue growing along the scaffold and at scaffold intersections, regions of interest (ROI, *n* = 5) were acquired first from bright-field microscopy images of the tissue growing on the scaffold. For measurements of tissue thickness along the scaffold, 4 measurements were taken from each ROI (*n* = 20 measurements) for each time point. For measurements of tissue thickness at scaffold intersections, the maximum thickness of the tissue at the scaffold intersection was measured for each ROI (*n* = 5 measurements). Statistical analysis was performed using Welch’s *t*-test, due to the unequal sample sizes between groups.

### Time-Lapse Imaging

For time-lapse imaging, scaffolds were placed in glass bottom 6 well plates (Ibidi) containing fresh media. The Scaffold-CellCrown assembly was lifted ≈1 mm from the bottom of the surface. SiR-Actin (Cytokeleton, Inc.) (100 nm) was added to the mTESR media ≈1 h before imaging. A fully automated CellDiscoverer 7 microscope with Zen Blue software (Zeiss) was used to acquire images over time without correcting for drift. Cells were incubated in a microscope-mounted environmental chamber, which was maintained at 37 °C, 5% CO_2_ using the accompanying Zen Blue 2 software and hardware control systems.

### Time-Lapse Imaging Coated Scaffolds

For time-lapse imaging at days 1–5, seven regions of interest were imaged every 3 h in bright-field and fluorescence mode. Each position was acquired as a *z*-stack. On day 3, image acquisition was paused for ≈30 min as the well plate was removed from the microscope for media exchange. Imaging was resumed until day 5.

### Bright-Field Heatmaps

89 bright-field images of scaffold regions on day 5 from 2 independent experiments were concatenated to create a stack. Images were rotated if required to ensure that the scaffold fibers were aligned throughout the entire stack. A custom Fiji macro was used to overlay the images and create a heatmap, using the “fire” LUT.^[27]^

### Scanning Electron Microscopy of Cellularized Scaffolds

On day 5, samples were fixed in 4% (v/v) PFA in PBS at 4 °C overnight, followed by washing three times in PBS. To cut samples from the scaffold, the Scaffold-CellCrown assembly was placed on a sterile poly(dimethylsiloxane) (PDMS) cutting mat (with a thickness of ≈5 mm), and scaffold organoid samples were obtained using a 6 mm biopsy punch. Scaffold and tissue biopsy samples were then dehydrated in a series of 30, 50, 70, and 80% (v/v) ethanol in water for 30 min each. Then 90 and 100% (v/v) ethanol for 60 min each. Hexamethyldisilazane (HMDS) was added for 30 min, and the samples were left to dry overnight. Prior to imaging, samples were gold sputter coated using an Emitech K575X sputter coater for 30 s at a deposition current of 20 mA. Imaging was performed using a JEOL JSM-6400 SEM at 3 kV acceleration voltage in secondary electron (SE) mode.

### Wholemount Immunostaining of Scaffold Cultured EB Samples

At day 5, hESCs on scaffolds were fixed by incubation in 4% (v/v) PFA in PBS overnight at 4 °C and washed three times in PBS. Organoid samples were cut from the scaffold, using a PDMS cutting mat and a 6 mm biopsy punch. For wholemount staining, samples were left in a permeabilization and blocking solution (0.3% Triton X-100 and 5% BSA in PBS) for 2 h at room temperature. Primary antibodies were added to the permeabilization and blocking solution and left overnight at 4 °C. Antibodies are listed in Table S1 (Supporting Information). Samples were rinsed three times in PBS and then washed three times in PBS-T (PBS + 0.01% Triton X-100), before staining with secondary antibody or Phalloidin (Alexa Fluor) at a dilution of 1:250 for 2–3 h. Sections were washed twice in PBS-T, followed by a final wash in PBS.

### Cryosectioning Organoids

Organoids on scaffolds were fixed in 4% (v/v) PFA in PBS overnight at 4 °C and washed three times in PBS. To cut samples from the scaffold, the Scaffold-CellCrown assembly was placed on a sterile PDMS cutting mat, and scaffold organoid samples were obtained using a 6 mm biopsy punch. The organoid samples were transferred to a 30% (w/v) sterile filtered sucrose solution in PBS in a 6-well plate, using forceps, and left at 4 °C overnight. The sucrose solution was removed, and scaffold organoids were equilibrated in OCT (Optimal Cutting Temperature) embedding matrix (Sakura, cat no. 4583) for 10 min. Using forceps, samples were transferred to a cryomold placed on dry ice, and filled with OCT embedding matrix. Embedded scaffold organoid blocks were then stored at −80 °C until cryosectioning. Tissue slices (25 μM thick) were obtained using a cryostat (Cryostar NX70 Thermo Cryostat) at −12 °C and collected on Superfrost Ultra Plus slides.

### Immunostaining Organoid Sections

Organoid sections were washed in PBS (Dulbecco’s phosphate buffered saline, Gibco, cat. no. 14190-144), then permeabilized and blocked in 0.3% (v/v) Triton X-100 and 5% (w/v) BSA with 0.05% (v/v) sodium azide in PBS for 30 min at room temperature. Primary antibodies were added to the antibody solution (0.1% (v/v) Triton X-100, 5% (w/v) BSA, and 0.05% (v/v) sodium azide in PBS) and left in a humidified staining box overnight at 4 °C. Slides were rinsed three times in PBS then washed three times in PBS-T (0.01% (v/v) Triton X-100 in PBS), before staining with secondary antibody in the antibody staining solution at a dilution of 1:500 for 2 h in a dark humidified staining box at room temperature, followed by 10 min DAPI staining at 1:1000 dilution in antibody staining solution. Sections were washed twice in PBS-T for 15 min, then washed once in PBS for 15 min. Sections were then mounted with a coverglass using Fluorosave (Millipore).

### Confocal Imaging

Fluorescence images were acquired using an inverted Leica SP8 confocal microscope (Leica microsystems), or a Visiscope Spinning Disc Confocal (Visitron Systems GmbH) equipped with a Yokogawa W1 spinning disc. Fluorophores were excited with a 405-nm diode laser (DAPI), a 488 nm argon laser (GFP), a 543 nm HeNe laser (Alexa Fluor-543/555), and a 633 nm HeNe laser (Alexa Fluor-633/647).

### Raw Data Processing

Raw data was minimally processed using opensource image analysis software Fiji (version: 2.3.0). Where needed, maximum intensity z-projections were performed and colors (LUT) of individual channels for figure panels were adapted to fit color blindness regulations.

### Data Analysis of Scaffold-Grown Stem Cells–3D Segmentation of Lumens

For lumen segmentation, SOX2 and ZO1 stained tissues were imaged using confocal microscopy as *z*-stacks. The ZO1 marker was used to segment the lumen, using the Fiji plugin segmentation editor. The lumens were manually segmented using the segmentation editor on 4–5 slices, including the first and last slice, and the rest were interpolated. The segmented lumens were pseudo-colored in green and merged with the SOX2 channel, to create confocal cross-sections at the bisector angle. 3D segmentation images were made with a 3D script plugin in Fiji.^[27]^

### Lumen Volume

Lumen volume was calculated on the segmented lumen regions using the 3D objects counter in Fiji. 26 triangle-shaped scaffold regions were analyzed (*N* = 3 independent scaffolds, *n* = 26 different scaffold regions).

### Lumen Area and Circularity

Lumen area was determined by first generating a maximum intensity *z*-projection, and manually segmenting the lumen. The inbuilt measure function in Fiji was used to calculate area and circularity. Circularity was defined as 4*π*(area/perimeter^2^), with a value of 1 corresponding to a perfect circle and values approaching 0 corresponding to increasingly elongated polygons. 26 triangle-shaped scaffold regions were analyzed (*N* = 3 independent scaffolds, *n* = 26 different scaffold regions).

### Total Cell Count

The central 6 slices of the *z*-stack with the maximum tissue area were summed as a maximum intensity projection. A median smoothing filter was applied with a value of 1, and 0.5 gamma was applied. Images were processed by subtracting a rolling ball-based calculated background with a rolling ball radius of 5. For cell count, images were thresholded using Auto Huang, and outliers were removed. Adjustable watershed was then applied, and ROIs were drawn at 500 μM from the scaffold center for each angle segment. Cells were counted in each segment using analyze particles function. Twenty-six triangle-shaped scaffold regions were analyzed (*N* = 3 independent scaffolds, *n* = 26 different scaffold regions).

Angles between 37° and 54° were grouped and denoted as ≈45° and all angles between 86° and 93° were grouped and denoted as ≈90°. Welch’s *t*-test was used for statistical analysis in GraphPad Prism (Dotmatics).

### Data Analysis of Scaffold-Grown Organoids-Organoid Circularity and Maximum Diameter

Tiled images of the entire scaffold were acquired using the CellDiscoverer 7 microscope as previously described. Scaffold-cultured organoid diameter and circularity were calculated by creating 5 ROIs for each independent scaffold and using a custom-built macro in Fiji to segment the individual organoids by thresholding using the Auto Huang algorithm. The same custom-built macro was used to segment the control organoids. The inbuilt measure function in Fiji was used to calculate the area and circularity of the segmented organoid regions.

### Scaffold Organoid Occupancy

Scaffold occupancy was calculated by creating an ROI in the center of the scaffold, and the number of scaffold intersections was counted using the inbuilt Fiji plugin CellCounter. Organoid occupancy was measured by counting the number of occupied intersections in the ROI region on 3 independent scaffolds (*N* = 3 independent biological repeats) using the CellCounter plugin.

### Mathematical Modelling

A mathematical model based on discrete curve-shortening flow was implemented in Matlab (Matlab 2020b, Mathworks) to model the shape of the evolving tissue on the square, parallelogram, and triangular-shaped scaffolds. Briefly, the model took a specific polygon as input (representing the scaffold walls), which was discretized into a user-defined number of points *n*_p_ and an equal number of line segments. Subsequently, the algorithm evolves each point in the normal direction toward the center of the original polygon at a speed that was proportional to the local line curvature, and this process was repeated for a number of iterations *j*_total_. For every point *p_i_* with neighbouring points *p*_*i*–1_ and *p*_*i*+1_, the normal vector **n** is computed as:

(1) n=∥u∥⋅v+∥v∥⋅u

where **u** = *p*_*i*+1_ − *p_i_*, **v** = *p*_*i*–1_ − *p*_*i*_, and || · || represents the vector norm. The local curvature (*k_i_*) at every point was computed using the LineCurvature2D Matlab function, which locally fitted a polygon to the points and calculated the curvature of the polygon analytically.^[28]^ For any iteration *j* < *j*_total_, the evolution of any point *p_i_* is calculated as:

(2) $$p_{i}^{j+1}=p_{i}^{j}+\delta n_{i}k_{i}$$

where *δ* is a user-defined control parameter to tune the rate at which the polygon shape evolved inward so that it could match the experimental observations. Thus, for every new time step (*j* + 1), the points from the previous time step (*j*) moved inward in the normal direction (**n**) at a speed that was determined by their local curvature (*k_i_*) and the control parameter (*δ*). After every evolution, the points along the evolved polygon shape were uniformly remeshed using the interparc Matlab function. Moreover, the first and last points along the evolving polygon shape were collapsed when they moved too close to each other, that was when their distance was within 5% of their original distance. This was done to avoid numerical errors in the curvature calculation, which would lead to locally very high (and unstable) speeds in the curve-shortening flow algorithm. To show the evolution of the initial polygon shape, representing the evolution of the tissue–medium interface, the evolved polygon points were plotted at different iterations *j*. Moreover, the normalized line curvature $\tilde{k}$ for different iterations was also plotted, with $\tilde{k}=n\cdot k/\left(k_{\max}^{j=1}-k_{\min}^{j=1}\right)$.

### Statistical Analysis

The biological and technical repeats were stated in the figure captions. The capital letter *N* designated independent biological repeats (i.e., independent scaffolds placed in a separate well) while the lowercase *n* designated technical repeats (i.e., different regions on the same scaffold). For statistical analysis of scaffold tensile modulus, the unpaired *t*-test was used, with a 95% confidence interval (GraphPad Prism 9, Version 9.2.0, GraphPad Software, Inc.). For analysis of interconnected EBs and organoids on scaffolds Welch’s *t*-test was employed, with a 95% confidence interval (GraphPad Prism 9, Version 9.2.0, GraphPad Software, Inc.). For statistical analysis of spatially separated organoids on scaffolds, the Kruskal–Wallis test with Dunn’s post-test for multiple comparisons was used, with a 95% confidence interval. Statistical significance was considered for all comparisons with *p* < 0.05.

## Supplementary Material

Supplementary material

## Data Availability

The data that support the findings of this study are openly available in Zenodo at http://doi.org/10.5281/zenodo.8221826.
