# Supplementary material for: Microfibrous Scaffolds Guide Stem Cell Lumenogenesis and Brain Organoid Engineering
Source: Adv Mater. Author manuscript; Available in PMC 2024 Dec 9. (PMC7617127; doi:10.1002/adma.202300305)
Supplement: Supplementary material [file EMS193196-supplement-Supplementary_material.pdf]

ADVANCED MATERIALS

Supporting Information

for *Adv. Mater.*, DOI 10.1002/adma.202300305

Microfibrous Scaffolds Guide Stem Cell Lumenogenesis and Brain Organoid Engineering

Kaja I. Ritzau-Reid, Sebastien J. P. Callens, Ruoxiao Xie, Martina Cihova, Daniel Reumann, Christopher L. Grigsby, Lino Prados-Martin, Richard Wang, Axel C. Moore, James P. K. Armstrong, Juergen A. Knoblich and Molly M. Stevens**

Supporting information

Microfibrous scaffolds guide stem cell lumenogenesis and brain organoid engineering

Kaja I. Ritzau-Reid[1], Sebastien J. P. Callens[1], Ruoxiao Xie, Martina Cihova, Daniel Reumann, Christopher L. Grigsby, Lino Prados-Martin, Richard Wang, Axel C. Moore, James P. K. Armstrong, Juergen A. Knoblich, Molly M. Stevens.**

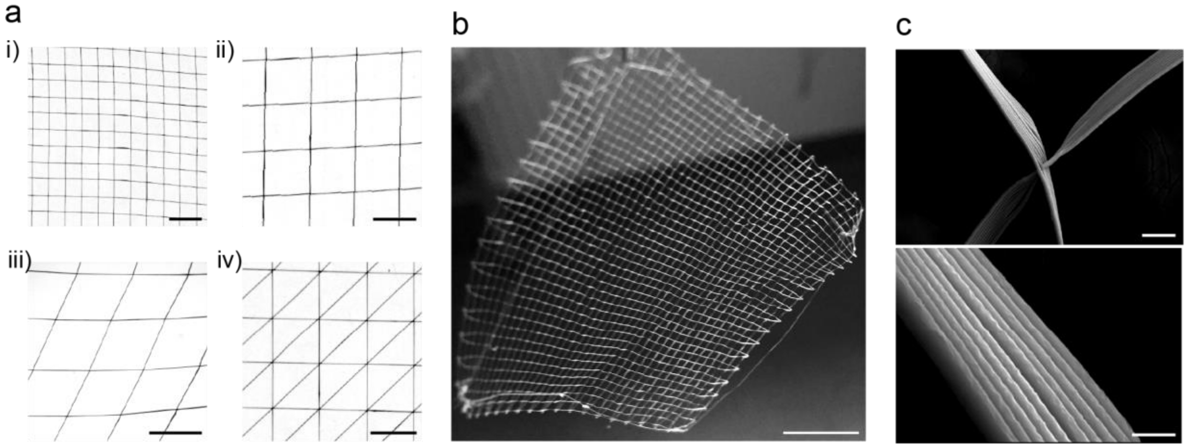

Supplementary Figure 1. Fabrication of scaffolds using MEW. a) Brightfield images of different MEW scaffold geometries, including: i) square grid scaffolds with 500 µm spacing, ii) square grid scaffolds with 1000 µm spacing, iii) rhombus grid scaffolds with 1000 µm spacing, iv) triangle grid scaffolds. Scale bar corresponds to 500 µm. b) Photograph of a PCL scaffold fabricated by MEW. Scale bar corresponds to 1 cm. c) SEM images of scaffolds with a square grid geometry. Scale bar corresponds to 50 µm (top) and 10 µm (bottom).

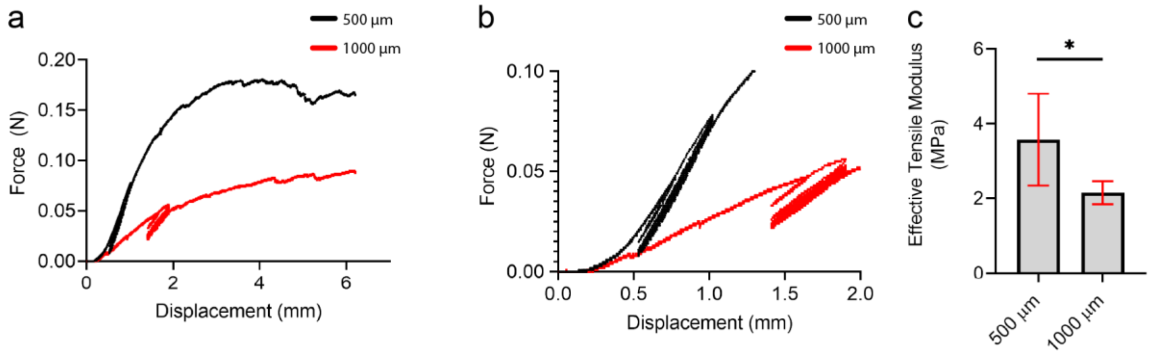

Supplementary Figure 2. Mechanical characterization of MEW scaffolds. a-b) Force-displacement curves of square grid scaffolds with 500 µm and 1000 µm spacing. c) Mean effective tensile modulus of square grid scaffolds of 500 µm and 1000 µm spacing (N = 5). Data represent mean ± s.d. * $p < 0.05$, determined by unpaired t-test.

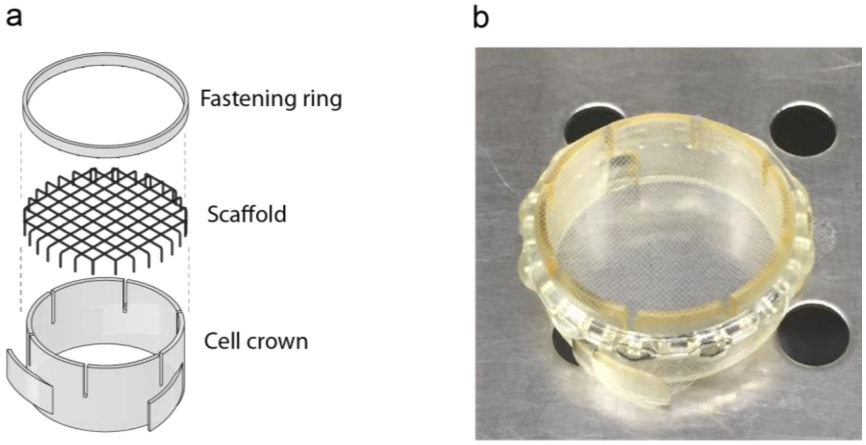

Supplementary Figure 3. S**caffold preparation for cell culture.** a) Schematic of cell crown and scaffold assembly. b) Photograph of a PCL scaffold fabricated by MEW and assembled in cell crown holder.

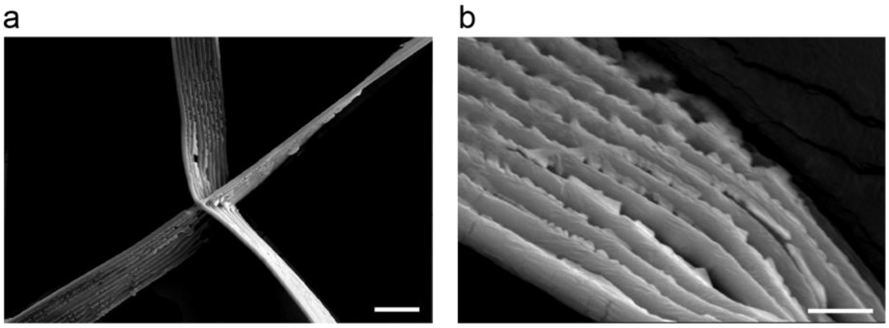

Supplementary Figure 4. SEM images of scaffolds coated with Matrigel basement membrane extract. Scale bar corresponds to left: 50 µm, right: 10 µm.

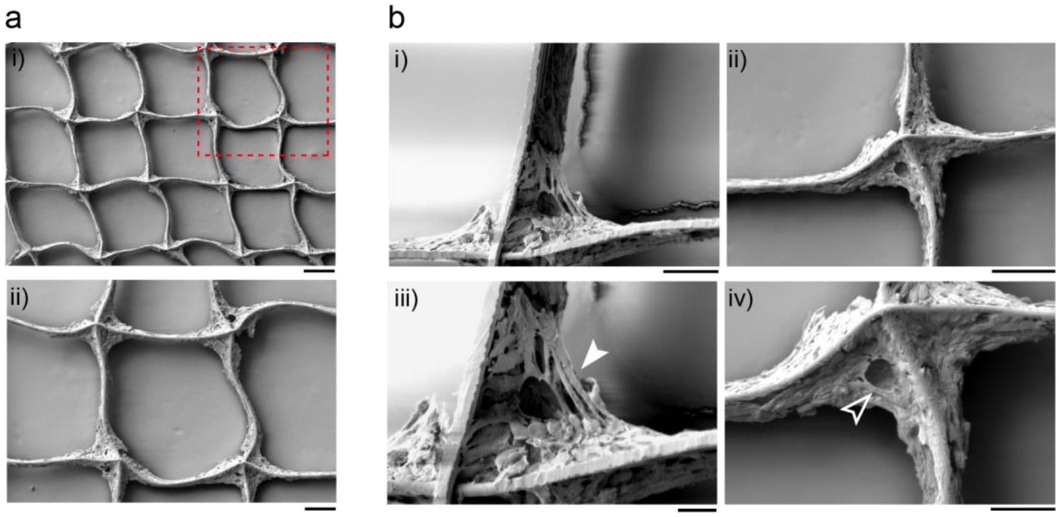

Supplementary Figure 5. SEM characterization of EB tissues on MEW. a) Representative SEM images of EB tissue on PCL scaffolds at day 5. Scale bar corresponds to 200 µm (i) and 100 µm (ii). b) High magnification images show elongated cells at the growing edge of the scaffold corner (solid white arrowhead) and the indent in the tissue of the lumen at the corner of the scaffold (empty white arrowhead). Scale bars correspond to (i) 50 µm, (ii) 100 µm, (iii) 20 µm, (iv) 50 µm.

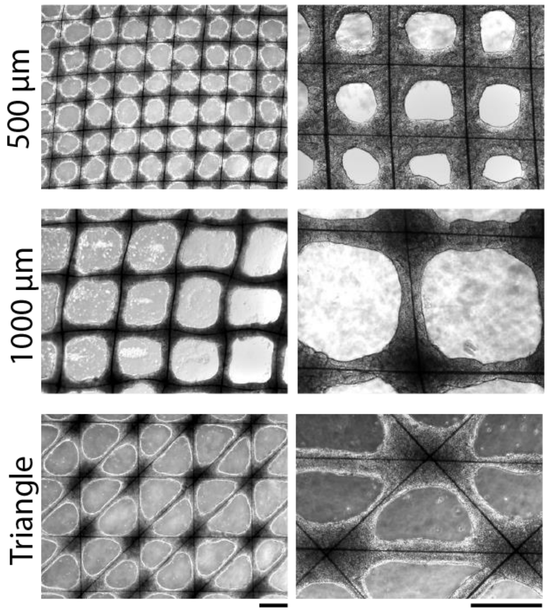

Supplementary Figure 6. Cerebral organoids on scaffolds at day 17. Representative brightfield images showing the development of cerebral organoids on scaffolds at day 17 on square grid scaffolds of 500 µm spacing, square grid scaffolds of 1000 µm spacing, and on triangle grid scaffolds. Scale bars corresponds to 500 µm.

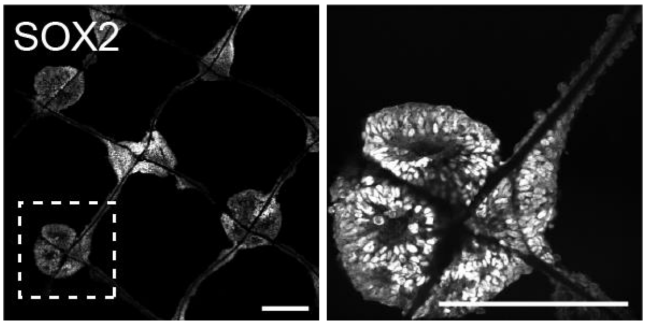

Supplementary Figure 7. Histological sections of organoids at day 20 immunostained with the neural progenitor marker SOX2. The image on the left shows an overview of multiple organoids and the image on the right shows a magnified view of the respective region marked with a white dashed line (representative images from 1 or 2 independent scaffolds). Scale bars correspond to 200 µm.

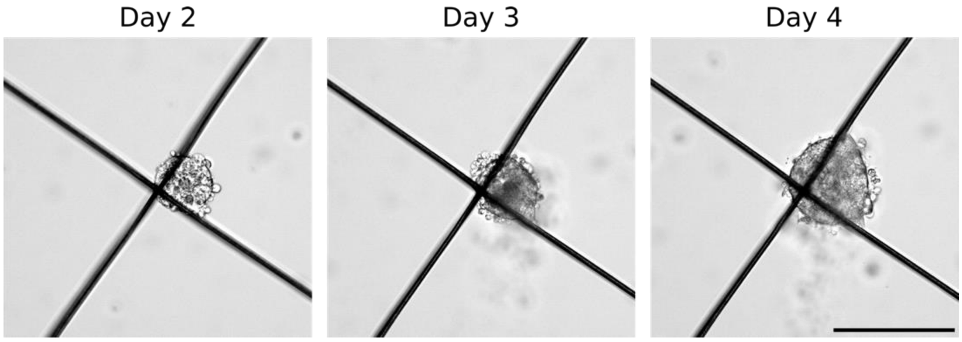

Supplementary Figure 8. EB tissue formation at scaffold intersections. Representative brightfield time-lapse images at Day 2-4 show hESCs aggregate and grow EB tissue at the scaffold intersection. 7 positions were imaged on one scaffold (N = 1, n = 7). Scale bar corresponds to 200 µm.

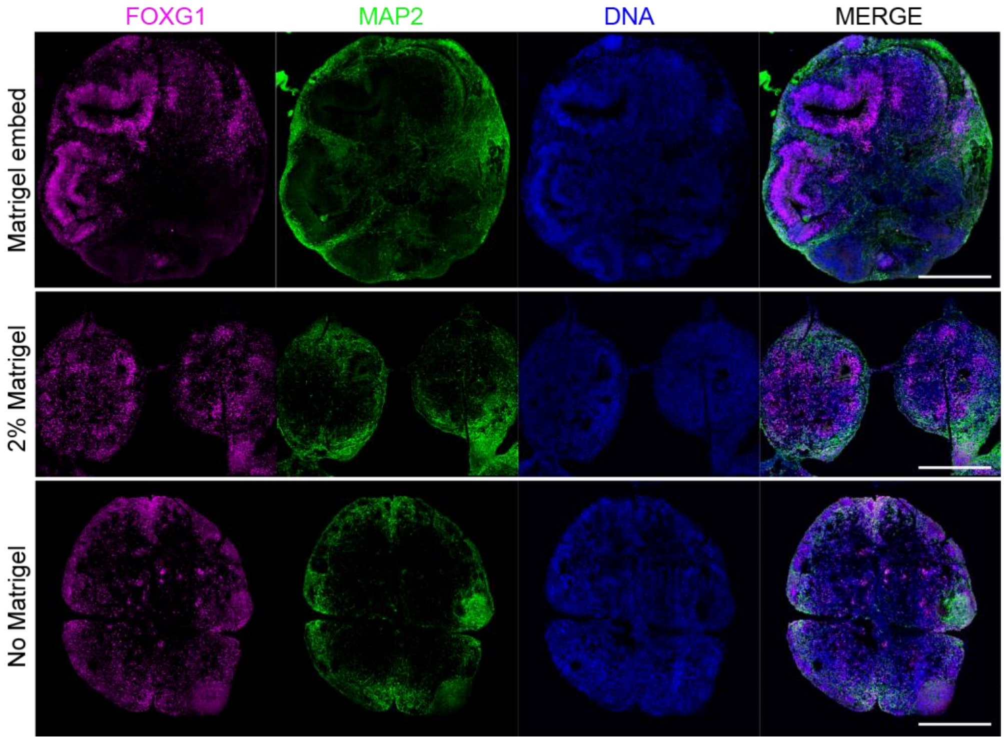

Supplementary Figure 9. Matrigel embedding optimization. Image gallery showing representative images from histological sections of organoids on scaffolds from different treatments: Matrigel embedding (top row), supplementation with 2% Matrigel (middle row) and a control group without Matrigel. Sections were immunostained with forebrain marker FOXG1 (magenta), neuronal marker MAP2 (green) and counterstained with DAPI (blue). (Representative images from a minimum of 2 independent scaffolds). Scale bar corresponds to 500 µm.

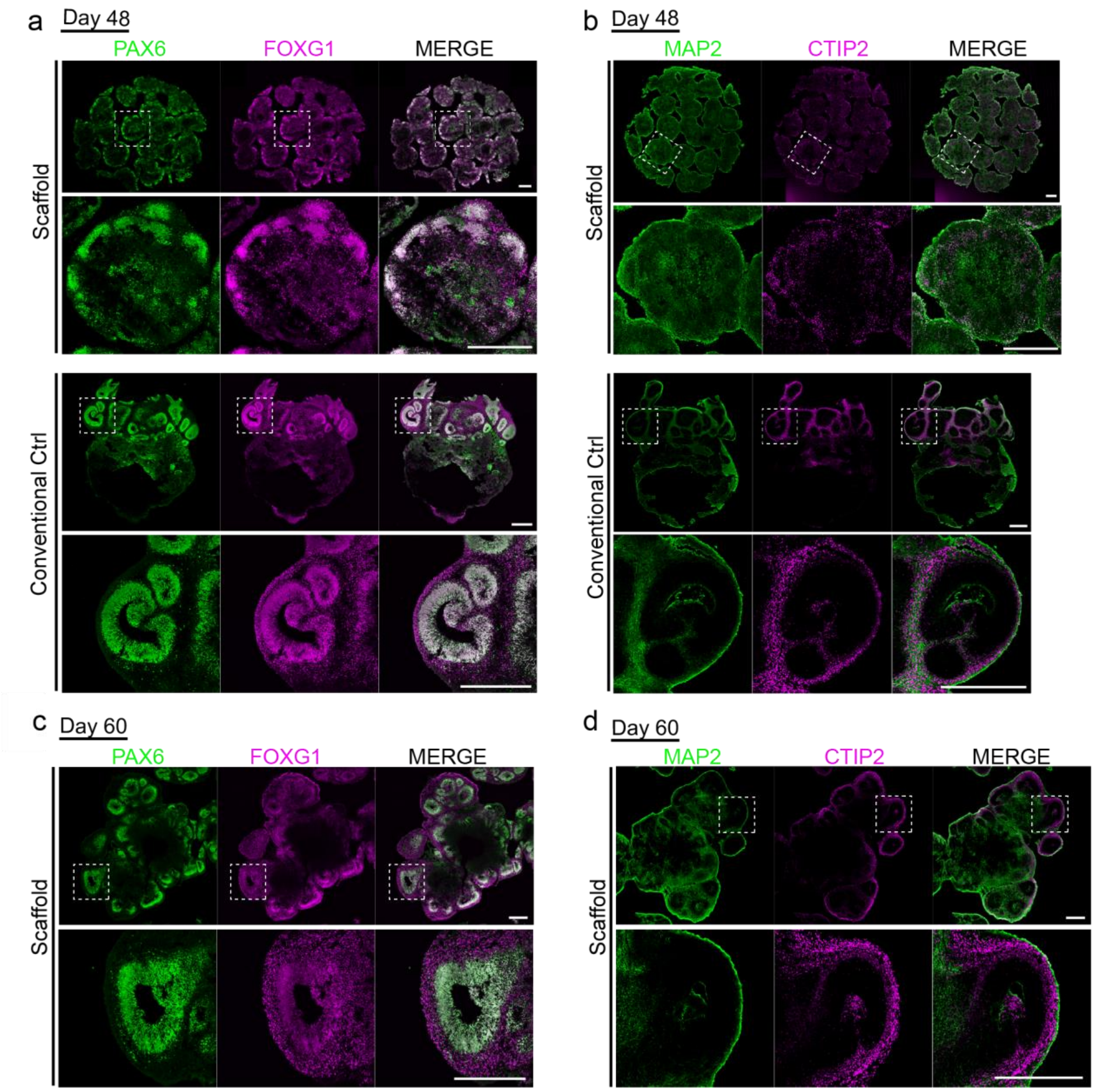

Supplementary Figure 10. Immunostaining characterization of long-term cerebral organoid culture on scaffolds. a) Image gallery of day 48 organoid histological sections immunostained with dorsal marker PAX6 (green) and forebrain marker FOXG1 (magenta) (Representative images from 1 scaffold). Scale bar corresponds to 500 µm. b) Image gallery of day 48 organoid histological sections immunostained with neuronal marker MAP2 (green), and deep cortical marker CTIP2 (magenta). Magnified regions are marked with a white dashed line (Representative images from 1 scaffold). Scale bar corresponds to 500 µm. c) Image gallery of day 60 scaffold organoid histological sections immunostained with dorsal marker PAX6 (green), and forebrain marker FOXG1 (magenta) (Representative images from 1 scaffold). Scale bar corresponds to 500 µm. d) Image gallery of day 60 organoid histological sections immunostained with neuronal marker MAP2 (green), and deep cortical marker CTIP2 (magenta). Magnified regions are indicated by white dashed line. (Representative images from 1 scaffold). Scale bar corresponds to 500 µm.

Supplementary Table S1 | Primary antibodies used.

Antigen	Host species	Supplier	Cat number	Dilution
SOX2	Rabbit	Abcam	ab97959	1:600
Z01	Moy use	Thermo Scientific	33-9100	1:50
NANOG	Rabbit	Abcam	ab21624	1:100
OCT4	Mouse	Abcam	ab184665	1:100
FOXG1	Rabbit	Abcam	ab18259	1:200
PAX6	Sheep	R&D Systabems	AF8150	1:200
aPKCzeta	Mouse	Santa Cruz	SC-17781	1:200
TUJ1	Mouse	Sigma-Aldrich	T8578	1:500
N-cadherin	Mouse	BD Biosciences	610920	1:500
GSX2	Rabbit	Sigma-Aldrich	ABN162	1:500
TBR1	Rabbit	Abcam	ab31940	1:300
MAP2	Chicken	Abcam	ab5392	1:500
CTIP2	Rat	Abcam	ab18465	1:300
